# Effect of Vitamin D Supplementation on *Mycobacterium tuberculosis*-Induced Innate Immune Responses in a Canadian Dené First Nations Cohort

**DOI:** 10.1371/journal.pone.0040692

**Published:** 2012-07-16

**Authors:** Linda Larcombe, Pamela Orr, Emily Turner-Brannen, Caroline R. Slivinski, Peter W. Nickerson, Neeloffer Mookherjee

**Affiliations:** 1 Department of Internal Medicine, University of Manitoba, Winnipeg, MB, Canada; 2 Manitoba Centre for Proteomics and Systems Biology, University of Manitoba, Winnipeg, MB, Canada; Colorado State University, United States of America

## Abstract

Canadian First Nations (FN) population experiences a high burden of tuberculosis. Vitamin D is known to enhance the expression of innate immune effectors, including cathelicidin LL-37, for protection against infections. In this study we performed longitudinal analyses to investigate the impact of vitamin D supplementation on macrophage responses to *Mycobacterium tuberculosis* (Mtb) lipoprotein (TLR2/1L), in Canadian Dené FN participants compared to Caucasian participants. Serum 25(OH)D and LL-37 levels were evaluated by ELISA. Transcriptional responses and protein expression of TLR2/1L-induced LL-37 and other innate immune cytokines were monitored in monocyte-derived macrophages (MDMs) before and after 8 months of vitamin D supplementation. In this study we showed that serum levels of LL-37 decreased after vitamin D supplementation in both Dené and Caucasian participants. There was no difference in TLR2/1L-induced LL-37 expression in MDMs in the two groups, either pre- or post-vitamin D supplementation. However, vitamin D supplementation markedly enhanced TLR2/1L-induced responses in MDMs e.g. IL-6, IL-12 and IL-23 among Caucasians but not in the Dené participants. In contrast, after vitamin D supplementation TLR2/1L-induced responses e.g. IL-1β, IL-8 and IL-12 were significantly reduced in the Dené MDMs. These results indicate that vitamin D supplementation enhanced TLR2/1L-induced innate immune macrophage responses in the Caucasian but not in the Dené participants. We hypothesize that cytokines may be differentially regulated in Canadian FN compared to Caucasians, in particular those that influence Th-1 and Th-17 responses required for the control of Mtb.

## Introduction

The incidence of tuberculosis (TB) is significantly higher among the Canadian First Nations (FN) populations compared to Caucasians [1] (www.phac-aspc.gc.ca). More than two decade ago it was demonstrated that vitamin D could inhibit *Mycobacterium tuberculosis* (Mtb) growth [2], and recently an association was demonstrated between vitamin D insufficiency and patients with TB [3]. Recent studies have demonstrated that vitamin D metabolites can promote innate immune responses required for the elimination of Mtb, largely by inducing the expression of human host defence peptide cathelicidin LL-37 [4], [5], [6], [7]. Lower level of vitamin D has been linked to lower expression of LL-37 in monocytes, thus contributing to higher susceptibility to TB in African-Americans [8]. Impaired expression of LL-37 is known to increase susceptibility to various infections [9], [10], [11]. Consistent with this, LL-37 has been demonstrated to be protective in various animal models of infections and sepsis [12], [13], [14], [15]. The biological function of LL-37 in controlling infections is suggested to be largely due to the immunomodulatory functions mediated by the peptide [16], [17], [18], which includes tissue repair, induction of innate immune responses, influencing the differentiation and polarization of dendritic cells and T-cells, and autophagy [13], [17], [19], [20], [21], [22]. The gene encoding for LL-37 is a direct target of the vitamin D/vitamin D receptor complex [8], [23], [24]. Thus it may be hypothesized that vitamin D insufficiency may result in decreased expression of LL-37 and impaired immune responses to Mtb, contributing to increased susceptibility to TB.

Function of the vitamin D-LL-37 axis in immune responses to Mtb has not been investigated among Canadian FN populations. A previous study showed that Canadian Dené and Cree FN have a higher frequency of single nucleotide polymorphisms (SNPs) associated with low expression of vitamin D receptor (VDR) and interferon-γ (IFN-γ), potentially contributing to increased risk of TB disease [1]. A recent study has also shown that cellular regulation of lymphocytes mediated by killer immunoglobulin-like receptors may be different in Canadian Oji-Cree FN compared to Caucasians, which in turn can contribute to differential outcome to infectious challenge [25]. These findings suggest that although social and environmental risk factors for disease contribute greatly to the increased burden of morbidity and mortality associated with TB in Canadian FN populations, underlying immune responses if differentially regulated may also play a role [1], [25], [26], [27]. We are engaged in a participatory research partnership with the Dené FN community of Lac Brochet in northern Manitoba, Canada, in order to elucidate the biologic and social determinants of TB, a disease that remains endemic among their people. There is no legal definition for the term “First Nations”, but it may be understood to mean a band within the meaning of the Canadian Indian Act, which includes Dené, Cree, Ojibwa and Oji-Cree. The Dené are part of the larger Na-Dene (Athapaskan) language family which include Alaskan Gwich’in and the American Apache and Navaho peoples. The Denesuline are a distinct group of Dené.

In this study we investigated the impact of vitamin D supplementation on the induction of cathelicidin host defence peptide LL-37 and innate immune cytokines and chemokines following stimulation of macrophages with a lipoprotein antigen (TLR2/1L) derived from Mtb [8], [28], in participants from the Canadian Dené community compared to Caucasians. Overall, we showed that there was no difference in TLR2/1L-induced LL-37 expression (both gene and protein level) in macrophages in the two groups. We showed that even though vitamin D supplementation markedly enhanced TLR2/1L-induced responses in macrophages e.g. IL-6, IL-12 and IL-23 among Caucasians, it did not have that effect in the Dené FN participants. In fact, TLR2/1L-induced innate immune responses such as IL-1β, IL-8 and IL-12 were significantly reduced in the Dené participants after vitamin D supplementation.

## Results

### Mtb Lipopeptide-induced LL-37 Expression in Macrophages

In this study we stimulated monocyte-derived macrophages (MDMs) with a 19 kDa lipopeptide derived from Mtb (TLR2/1L), which is known to be a ligand for Toll-like receptor (TLR) [28]. Previous studies have demonstrated that Mtb lipopeptide TLR2/1L-mediated activation of TLR2/1 triggers effector antimicrobial innate immune responses that confer protection against Mtb [8], [28], [29]. It has also been shown that TLR2/1L stimulation of macrophages results in the induction of LL-37 and other innate immune responses, in the presence of autologous sera as a source of vitamin D [8]. Therefore, in this study, prior to evaluating TLR2/1L-induced responses in MDMs we evaluated serum levels of 25(OH)D as a determinant of vitamin D status [30]. In a recent study we have shown that serum concentrations of 25(OH)D is significantly lower in winter in the Canadian Dené population compared to Caucasians, whereas summer levels are similar between the two groups (Larcombe et al, manuscript submitted). Therefore, for this study we collected blood samples in late summer/fall (summer levels), both pre- and post-vitamin D supplementation, to eliminate seasonal differences. There was no significant difference in the amount of serum 25(OH)D between the Dené and Caucasian participants, both pre- and post-vitamin D supplementation (Table 1). Mean serum 25(OH)D was >75 nmol/L in both the groups, pre- and post-vitamin D supplementation (Table 1). 75 nmol/L 25(OH)D is the consensus serum level minimally required for prevention of diseases [30]. There was no significant difference in serum LL-37 levels between the Dené and Caucasians participants, pre- and post-vitamin D supplementation (Table 1). However, after 8 month of vitamin D supplementation the serum concentrations of LL-37 decreased more than 2-fold (p = 0.008) in the Dené and ∼1.3-fold (p = 0.03) in the Caucasian participants (Table 1).

**Table 1:** Circulating serum levels of 25(OH)D and LL-37.

	Pre-vitamin D supplementation		Post-vitamin D supplementation	
	25(OH)D	LL-37	25(OH)D	LL-37
**Dené** (n = 5)	109±40 nmol/L	133±54 ng/ml	97±36 nmol/L	58±23 ng/ml
**Caucasians** (n = 5)	89±24 nmol/L	170±78 ng/ml	98±33 nmol/L	123±23 ng/ml

Results shown are average ± standard deviation.

**Figure 1:**
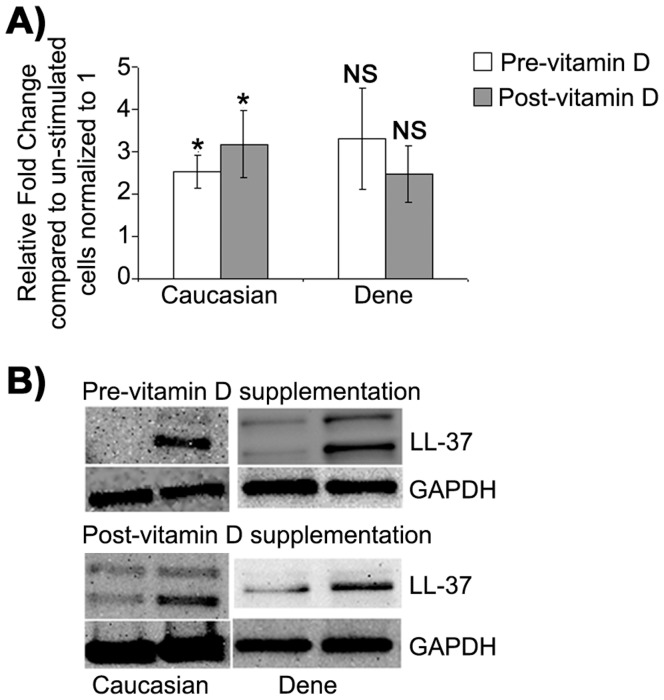
TLR2/1L-induced expression of human host defence peptide LL-37 in macrophages. MDMs were stimulated with TLR2/1L (10 µg/ml) in the presence of autologous serum. (**A**) Gene expression of LL-37 was monitored by qRT-PCR after 6 hr. Fold changes (y-axis) was normalized to 18S RNA and is represented relative to gene expression in un-stimulated cells normalized to 1 using the comparative Ct method. Results represent an average of five independent experiments (MDMs isolated from five Dené and five Caucasian participants) ± standard error (*p<0.05 and NS =  non-significant). (**B**) LL-37 peptide expression was monitored by probing immunoblots with anti-LL-37 antibody after 21 hr. The immunoblot shown is a representative blot of 4 independent experiments from MDMs isolated from independent Dené and Caucasian participants each.

In order to specifically evaluate induction of LL-37 peptide in macrophages, MDMs were stimulated with TLR2/1L in the presence of autologous serum, mRNA expression of LL-37 was evaluated by qRT-PCR after 6 hr and peptide expression was evaluated by Western blots after 21 hr. LL-37 transcript was up-regulated in MDMs in the presence of the TLR2/1L by 2.5-fold (p = 0.05) pre-vitamin D and by 3-fold (p = 0.03) after vitamin D supplementation in the Caucasians participants (Fig. 1A). Similarly, LL-37 mRNA was up-regulated between 2 to 3-fold both before and after vitamin D supplementation in the Dené participants however this induction was not statistically significant (Fig. 1A). There was no statistically significant difference in the up-regulation of LL-37 mRNA expression in the Dené compared to the Caucasian participants, either pre- or post-vitamin D supplementation (Fig. 1A). Similar trend was also seen at the protein level with Western blot analysis probing with anti-LL-37 antibody. LL-37 peptide was enhanced around 2-fold following stimulation with TLR2/1L in both groups (Fig. 1B) and there was no statistically significant difference in the amount of peptide induced between the two groups as measured by densitometry (data not shown). These results suggested that the ability of macrophages to induce host defence peptide LL-37 expression following stimulation with Mtb lipopeptide was not significantly different between the Canadian Dené and the Caucasian participants, and that the induction of LL-37 expression was not significantly altered after vitamin D supplementation in this study.

### Mtb Lipopeptide-induced Cytokine and Chemokine Transcriptional Responses

**Figure 2:**
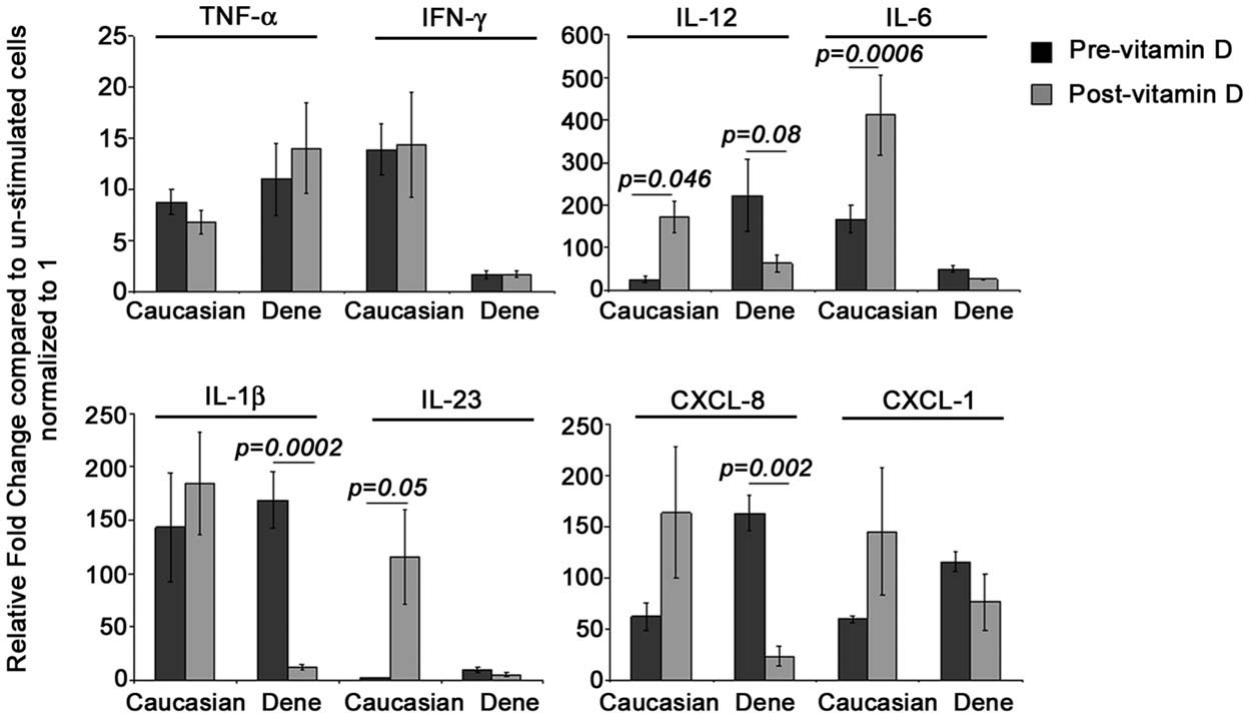
TLR2/1L-induced cytokine and chemokine transcriptional responses in macrophages. MDMs were stimulated with TLR2/1L (10 µg/ml) in the presence of autologous serum. Gene expression was monitored by qRT-PCR after 6 hr. Fold changes (y-axis) was normalized to 18S RNA and is represented relative to gene expression in un-stimulated cells normalized to 1 using the comparative Ct method. Results are average of at least four independent experiments (MDMs isolated from four to five independent donors each from the Dené and Caucasians participants) ± standard error.

Innate immune cytokines are critical in the control of Mtb infection, efficient polarization of adaptive immunity, as well as in limiting granuloma in TB [31]. We evaluated TLR2/1L-induced innate immune cytokines e.g. TNF-α, IL-1β, IL-6, IL-23 and IL-12, known to be critical players in the control of Mtb infection, and chemokines such as Gro-α and IL-8 that are required for cell recruitment to the site of infection [31]. Pre-vitamin D supplementation, TLR2/1L-induced gene expression of IL-23 was 3-fold higher (p = 0.04) and IL-12 was 10-fold higher (p = 0.07) in MDMs from the Dené compared to the Caucasian participants (Fig. 2). In contrast, TLR2/1L-induced gene expression of IL-6 was 3-fold less (p = 0.01) and IFN-γ was 8-fold less (p = 0.03) in MDMs from the Dené compared to the Caucasian participants pre-vitamin D supplementation (Fig. 2). There was no significant difference between TLR2/1L-induced cytokines TNF-α and IL-1β between the two groups, whereas gene expression of chemokines IL-8 (encoded by CXCL8) and Gro-α (encoded by CXCL1) was modestly (around 2-fold) higher in MDMs from the Dené participants (p<0.05) compared to Caucasians, pre-vitamin D supplementation.

In order to evaluate the effect of vitamin D supplementation on TLR-2/1L-induced responses in macrophages, cytokine and chemokine gene expressions were evaluated after 8 months of vitamin D supplementation of the participants in this study. Post-vitamin D supplementation there was a marked increase in TLR2/1L-induced transcriptional responses in MDMs from the Caucasian participants; 48±18-fold increase (p = 0.05) in IL-23, 9±2-fold increase (p = 0.014) in IL-12 and 4±1-fold increase in IL-6 (p = 0.0006) (Fig. 2). In contrast, post-vitamin D supplementation there was no significant enhancement of TLR2/1L-induced gene expression of either IL-6 or IL-23 in macrophages from the Dené participants compared to gene expression before vitamin D supplementation (Fig. 2). Also, in contrast to the Caucasian MDMs, TLR2/1L-induced gene expression of IL-12 decreased by 6±2-fold (p = 0.08), IL-1β decreased by 16±3.6-fold (p = 0.0002) and IL-8 (CXCL8) decreased by 12±4-fold (p = 0.002) after vitamin D supplementation in MDMs from the Dené participants (Fig. 2).

### Mtb Lipopeptide-induced Protein Production of IL-6 and Gro-α in Macrophages

**Figure 3:**
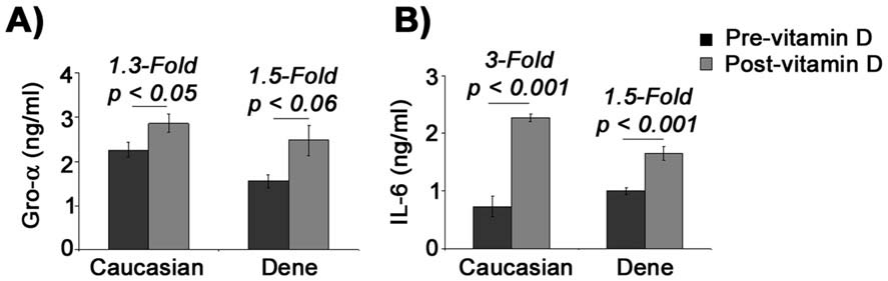
Protein production following stimulation with TLR2/1L. MDMs were stimulated with TLR2/1L (10 µg/ml) for 21 hr. TC supernatants were monitored for the production of (**A**) chemokine Gro-α and (**B**) cytokine IL-6 by ELISA. Results are shown after subtraction of background levels of Gro-α or IL-6 monitored in un-stimulated cells. All results are average of at least four independent experiments (MDMs isolated from four to five independent donors each from the Dené and Caucasians participants) ± standard error.

Consistent with gene expression results (Fig. 2), there was no statistically significant difference in the TLR2/1L-induced production of chemokine Gro-α (encoded by the gene CXCL1) in MDMs from the Dené compared to the Caucasian participants, both pre- and post-vitamin D supplementation (Fig. 3A). Production of Gro-α protein was modestly enhanced by approximately by 1.5-fold both in the Caucasian and Dené participants after vitamin D supplementation (Fig. 3A). Prior to vitamin D supplementation the amount of IL-6 detected in TC supernatants of un-stimulated MDMs was 10-fold less (p<0.05) in Caucasians (3.5±0.5 pg/ml) compared to the Dené participants (48±14 pg/ml), the baseline levels of IL-6 protein in un-stimulated MDMs increased more than 6-fold (*p*<0.05) post-vitamin D supplementation in Caucasians, while there was no significant change in the baseline levels of IL-6 in the Dené participants (data not shown). These results indicated that vitamin D supplementation increased the constitutive level of macrophage IL-6 production in Caucasians, but not in the Dené participants. Consistent with the gene expression data (Fig. 2), TLR2/1L-induced protein production of the cytokine IL-6 from MDMs was markedly enhanced by 3-fold (p = 0.0008) in the Caucasian participants after vitamin D supplementation compared to the amount of TLR2/1L-induced IL-6 production pre-vitamin D supplementation (Fig. 3B). Whereas, production of IL-6 in TLR2/1L-stimulated MDMs modestly increased by 1.5-fold (p = 0.0006) in the Dené participants after vitamin D supplementation (Fig. 3B). Thus vitamin D supplementation did not markedly enhance TLR2/1L-induced macrophage IL-6 protein production in the Dené participants, in contrast to that seen in the Caucasian participants.

## Discussion

Ability of vitamin D to control infections and autoimmunity is rapidly emerging as a new concept in disease management. The molecular mechanisms of immune-regulatory role of vitamin D in protecting against pathogens are not yet delineated. Implication of vitamin D as an anti-infective agent modulating host immune responses has largely been based on *in-vitro* studies [32]. Studies with various mouse models of infections to evaluate the impact of vitamin D on infection clearance have been ambivalent [32]. Vitamin D was demonstrated to be essential for IFN-γ-induced responses in monocytic cells which includes conversion of 25(OH)D to active 1,25(OH)_2_D_,_ activation of VDR, induction of host defence peptides i.e. cathelicidin and defensins, autophagy and phagosome maturation, collectively contributing to the control of TB [33]. Stimulation of macrophages with TLR2/1L derived from Mtb induces the expression of the VDR and the enzyme CYP27B1 (that catalyzes the conversion of 25(OH)D to active 1,25(OH)_2_D) and shown to inhibit Mtb *in-vitro* largely by the induction of innate immune responses including induction of cathelicidin host defence peptide LL-37 [8], [34]. Consistent with this, insufficient vitamin D status was recently attributed to impaired ability to mount a robust immune responses to Mtb among African-Americans [33]. Therefore, in this study we investigated the impact of vitamin D supplementation on Mtb-induced innate immune responses in members of a Dené FN community which has experienced high rates of TB for many years (www.phac-aspc.gc.ca).

We demonstrated that there was no significant difference in the summer circulating serum level of either 25(OH)D or LL-37 between the Dené and the Caucasian participants, both pre- and post-vitamin D supplementation (Table 1). Interestingly, it was noted that the serum concentrations of LL-37 was suppressed significantly in both the groups following vitamin D supplementation (Table 1). A recent study has demonstrated that vitamin D status does not correlate with the serum concentrations of LL-37 [3]. Cathelicidin LL-37 has been shown to have both pro- and anti-inflammatory properties which are cell/tissue type dependent, LL-37 can be both synergistic and antagonistic with other immune mediators and the expression of this peptide is known to be regulated by the vitamin D pathway [35]. Serum level of LL-37 is elevated in inflammatory conditions such as psoriasis and it has been suggested that LL-37 may be both an effector and regulator of inflammation [36]. Our results demonstrating that vitamin D supplementation in humans suppressed LL-37 concentration in serum indicates that vitamin D may play a role in balancing inflammation. This is consistent with previous studies that have demonstrated that the bioactive metabolite of vitamin D, 1,25(OH)_2_D, induces CD4+CD25+ T-reg cells (which mediates tolerance to transplantation and suppress autoimmune diabetes), suppress the production of Th-1/Th-17 cytokines e.g. IFN-γ and IL-17, and can suppress several Th-1/Th-17 inflammatory autoimmune diseases such as arthritis, lupus and inflammatory bowel disease [37], [38]. In this study there was no difference in TLR2/1L-induced expression of LL-37 in macrophages between Caucasians and the Dené participants, before or after vitamin D supplementation (Fig. 1). Overall the results in this study indicate that circulating serum levels of LL-37 decreases with vitamin D supplementation and that the regulation of LL-37 expression appears to be similar in the Dené and the Caucasians participants. Therefore, dysregulation of the cathelicidin host defence peptide LL-37 does not appear to be a contributing factor to the higher incidence of TB among the Dené FN.

The impact of vitamin D supplementation on Mtb-induced innate immune cytokine and chemokine responses in macrophages (the natural host of mycobacterium) was explored in this study. Our results suggest that even though the bioavailability of 25(OH)D was not different between the Dené and Caucasian participants (Table 1), Mtb-induced certain innate immune cytokine responses were differentially expressed in the Dené participants compared to Caucasians, and that supplementation with vitamin D did not enhance cytokine responses in the Dené participants (Fig. 2). We showed that macrophage responses to the Mtb lipoprotein (TLR2/1L) such as IL-6, IL-12 and IL-23, were markedly enhanced in Caucasians after vitamin D supplementation but not in the Dené participants (Fig. 2 and Fig. 3). Further, TLR2/1L-induced IL-1β, IL-12 and IL-8 were significantly reduced in the Dené macrophages and not Caucasians after vitamin D supplementation (Fig. 2). Cytokines that influence the polarization of Th-1 and Th-17 responses i.e. IFN-γ, IL-6, IL-12 and IL-23 were significantly lower in the Dené group compared to the Caucasians participants, post-vitamin D supplementation (Fig. 2). These results imply that either the vitamin D metabolic pathway or macrophage functions or both may be impaired among the Dené compared to the Caucasians participants.

Macrophages express the vitamin D receptor (VDR), CYP27B1 a critical enzyme in the vitamin D metabolic pathway and target genes known to be induced by the VDR-complex [5], [39]. The enzyme CYP27B1 converts 25(OH)D to the active metabolite 1,25(OH)_2_D, which acts in an intracrine mechanism by interacting with VDR to induce the expression of innate immune responses in macrophages to control bacterial challenge [8], [34], [40]. As the substrate i.e. serum 25(OH)D was not different in the Dené group compared to the Caucasians (Table 1), we speculate the possibilities for reduced Mtb-induced cytokine responses among the Dené in this study as follows.

It is known that Mtb lipoprotein TLR2/1L induces the expression of VDR and CYP27B1 via TLR2/1-signalling in macrophages [40]. The TLR2/1-mediated signalling pathway may be dysregulated in the Dené, resulting in reduced expression of the enzyme CYP27B1, therefore resulting in suppressed innate immune gene expression. It has been recently demonstrated that TLR2/1L-mediated induction of IL-1β is required for the up-regulation of antimicrobial effectors such as beta-defensin but not for induction of cathelicidin LL-37 [29]. It was also demonstrated that vitamin D activation and TLR2/1L-induced IL-1β signalling pathways in macrophages are distinct, and that both these pathways are required for the antimicrobial activity against Mtb [29]. This is consistent with the results in our study which demonstrated that TLR2/1L-induced LL-37 responses were not different (Fig. 1), whereas effects of vitamin D supplementation on TLR2/1L-induced IL-1β and other innate immune cytokine responses such as IL-6, IL-8, IL-12, IL-23 and IFN-γ were different in the Dené compared to Caucasians (Fig. 2 and Fig. 3). Recent studies have demonstrated that IL-1β-regulates host resistance against Mtb by mechanisms that is distinct from the TNF-α-dependent pathway [31], which is also consistent with this study’s findings that TLR2/1L-induced TNF-α gene expression was not different between the two groups, whereas induction of IL-1β was significantly reduced in the Dené post-vitamin D supplementation which was different from the response seen in the Caucasian participants (Fig. 2). Therefore, this study’s results suggests that the vitamin D activation pathway may not be significantly different between the Dené FN and Caucasians, however Mtb-induced TLR-signalling and resulting downstream cytokine responses may be differentially regulated in the Dené FN compared to the Caucasians, thus contributing to reduced antimicrobial functions and possibly to higher incidence of TB among the Dené. Delineating the TLR-mediated innate immune molecular mechanisms in macrophages among Canadian FN and other vulnerable populations may provide potential new preventive approaches either through defining alternate therapeutic targets or vaccines.

This study suggests that vitamin D supplementation may not result in enhancing immunity to infections, in particular for intracellular pathogens such as Mtb, in this Dené cohort. Innate immune cellular responses induced in response to infections, in particular intracellular pathogens, may be dysregulated in the Dené FN compared to Caucasians. Our study supports future research in host-pathogen interactions and innate immunity in the Canadian FN population. Identification of specific innate immune pathways that may be differentially expressed and functionally different will allow for a rational development of targeted interventions not only for infectious diseases but also for immune-mediated chronic disorders for the FN population.

## Materials and Methods

### Study Participants

Venous blood was collected from individuals who self identified as Dené and who are members of the Denesuline FN, located at 58° latitude in northern Manitoba, Canada. Blood was also collected from self-identifying Canadian Caucasians recruited from a convenience sample in Winnipeg, Manitoba. Informed written consent was obtained from all study participants. The study was approved by the community Chief and Council, and the University of Manitoba Ethics Review Board. Canadian Aboriginal research principles of ownership, control, access and possession (OCAP) were followed (The First Nations Principles of OCAP. Ottawa: First Nations Governance Centre; 2010. Available at: http://www.fnigc.ca/node/2). Participants were 18 years or above, able and willing to give consent, were not on any vitamin supplementation during the first year of the study, did not have clinical evidence of infections at the time of blood collection, were not first degree relatives of other enrolled individuals, were not on immunosuppressive medication and did not have known immunosuppressive medical condition. Blood samples were collected in late summer/fall (September - November), both pre (2010) and post (2011) vitamin D supplementation to eliminate seasonal differences. The study participants were given 1000 IU of vitamin D supplementation from January to September (in the second year of the study, 2011).

### Cell Isolation and Culture

Serum was separated and peripheral blood-derived mononuclear cells (PBMC) were isolated from blood samples as previously described [17]. Monocyte-derived macrophages (MDMs) were derived from PBMC as previously described [8], [41]. Briefly, PBMC (1×10^7^ cells/well in a 6-well tissue culture plate) were cultured in RPMI-1640 media (Gibco, Invitrogen, Canada) containing 2 mM L-glutamine and 1 mM sodium pyruvate, supplemented with 10% FBS and maintained in a humidified incubator at 37°C and 5% CO_2_. After 3 hr incubation non-adherent cells were removed and the adherent cells were further cultured in RPMI media supplemented with 100 ng/ml human recombinant M-CSF (eBioscience Inc, USA) for 7 days to generate MDMs [8], [41]. It was previously shown that MDMs require autologous human serum (as a source of vitamin D) to induce innate immune responses, including expression of LL-37, following stimulation with the 19 kDa lipoprotein antigen (TLR2/1L) derived from Mtb [8]. Therefore, prior to stimulation with TLR2/1L, the media was changed to RPMI supplemented with 10% autologous human serum (without FBS). MDMs were stimulated with 10 µg/ml TLR2/1L (EMC Microcollections, Germany) in the presence of autologous serum for either 6 or 21 hr [8], [28].

### ELISA

Tissue culture (TC) supernatants were centrifuged at 1500× g for 5–7 min to obtain cell-free samples and aliquots were stored at −20°C. Chemokines Gro-α and IL-8 was monitored by ELISA using specific antibodies (R&D Systems Inc.) as per the manufacturer’s instructions. Cytokines IL-23 and IL-6 were evaluated using antibodies from eBioscience, Inc., by ELISA as per the manufacturer’s instructions. The concentrations of the cytokines or chemokines were determined by establishing a standard curve with serial dilutions of the recombinant specific protein. Serum 25-hydroxyvitamin D (25(OH)D) was determined by ELISA (Immunodiagnostic Systems, Inc., USA) and LL-37 was evaluated by ELISA (Hycult Biotechnology, Netherlands) as per the manufacturer’s instructions [3].

### Western Blots

Total cell lysates were prepared in lysis buffer containing 10 mM Tris-HCl pH 7.5, 150 mM NaCl, 2 mM EDTA, protease inhibitor cocktail (Sigma-Aldrich) and 1% Triton X-100. The lysates were electrophoretically resolved on 4–12% NuPAGE® Bis-Tris gels (Invitrogen) and transferred to nitrocellulose membranes (Millipore, Canada). The membranes were blocked with TBST (20 mM Tris pH 7.5, 150 mM NaCl, 0.1% Tween 20) containing 3% fish gelatin (Sigma) and probed with either a rabbit anti-LL-37 polyclonal antibody (a gift from Dr. Terry Pearson, University of Victoria, Canada), or a purified rabbit anti-GAPDH (Cell Signaling Technology, Inc., USA) in TBST containing 1% fish gelatin. Affinity purified HRP-linked secondary antibodies were used for detection and the membranes were developed with the Amersham ECL detection system (GE Healthcare, Canada).

### Quantitative Real-time PCR (qRT-PCR)

RNA was isolated and subsequently analyzed for gene expression by qRT-PCR using SuperScript III Platinum Two-Step qRT-PCR Kit with SYBR Green (Invitrogen) in the ABI PRISM 7300 sequence detection system (Applied Biosystems). Fold changes were calculated using the comparative Ct method [42], after normalization using 18S RNA primers. The list of primers employed in this study is shown in Table 2.

**Table 2:** Summary of primers used for quantitative real-time PCR.

Gene	Forward primer	Reverse Primer
TNF-α	cagcctcttctccttcctgat	gccagagggctgattagaga
IL1-β	ctgtcctgcgtgttgaaaga	ttgggtaatttttgggatctaca
IL-4	agctgatccgattcctgaaa	gttggcttccttcacaggac
IL-6	caggagcccagctatgaact	gaaggcagcaggcaacac
IL-23	agcttcatgcctccctactg	ctgctgagtctcccagtggt
IL-12p40	aggtcttgtccgtgaagactcta	ccctgacattctgcgttca
IFN-γ	ggcattttgaagaattggaaag	tttggatgctctggtcatctt
CXCL-1 (Gro-α)	tcctgcatcccccatagtta	cttcaggaacagccaccagt
CXCL-8 (IL-8)	agacagcagagcacacaagc	aggaaggctgccaagagag
18S RNA	gtaacccgttgaaccccatt	ccatccaatcggtagtagcg
